# Supplementary material for: ARID1A loss derepresses a group of human endogenous retrovirus-H loci to modulate BRD4-dependent transcription
Source: Nat Commun. 2022 Jun 17;13:3501. doi: 10.1038/s41467-022-31197-4 (PMC9205910; doi:10.1038/s41467-022-31197-4)
Supplement: Supplementary file 1 — Supplementary Information [file 41467_2022_31197_MOESM1_ESM.pdf]

Supplementary Information

ARID1A loss derepresses a group of human endogenous retrovirus-H loci to modulate BRD4-dependent transcription

Chunhong Yu[1,*], Xiaoyun Lei[1,*], Fang Chen[1], Song Mao[1], Lu Lv[1], Honglu Liu[1], Xueying Hu[1], Runhan Wang[2], Licong Shen[1,3], Na Zhang[1], Yang Meng[2], Yunfan Shen[2], Jiale Chen[2], Pishun Li[1], Shi Huang[2], Changwei Lin[1,5], Zhuohua Zhang[1,2,4], Kai Yuan[1,2,4,6,#]

[1]Hunan Key Laboratory of Molecular Precision Medicine, Department of Oncology, Xiangya Hospital, Central South University, Changsha, Hunan, China.
[2]Hunan Key Laboratory of Medical Genetics, School of Life Sciences, Central South University, Changsha, Hunan, China.
[3]Department of Gynecology, Xiangya Hospital, Central South University, Changsha, Hunan, China.
[4]National Clinical Research Center for Geriatric Disorders, Xiangya Hospital, Central South University, Changsha, Hunan, China.
[5]Department of Gastrointestinal Surgery, The Third Xiangya Hospital, Central South University, Changsha, Hunan, China.
[6]The Biobank of Xiangya Hospital, Central South University, Changsha, Hunan, China.
[*]These authors contributed equally.
[#]Corresponding author: yuankai@csu.edu.cn (K.Y.)

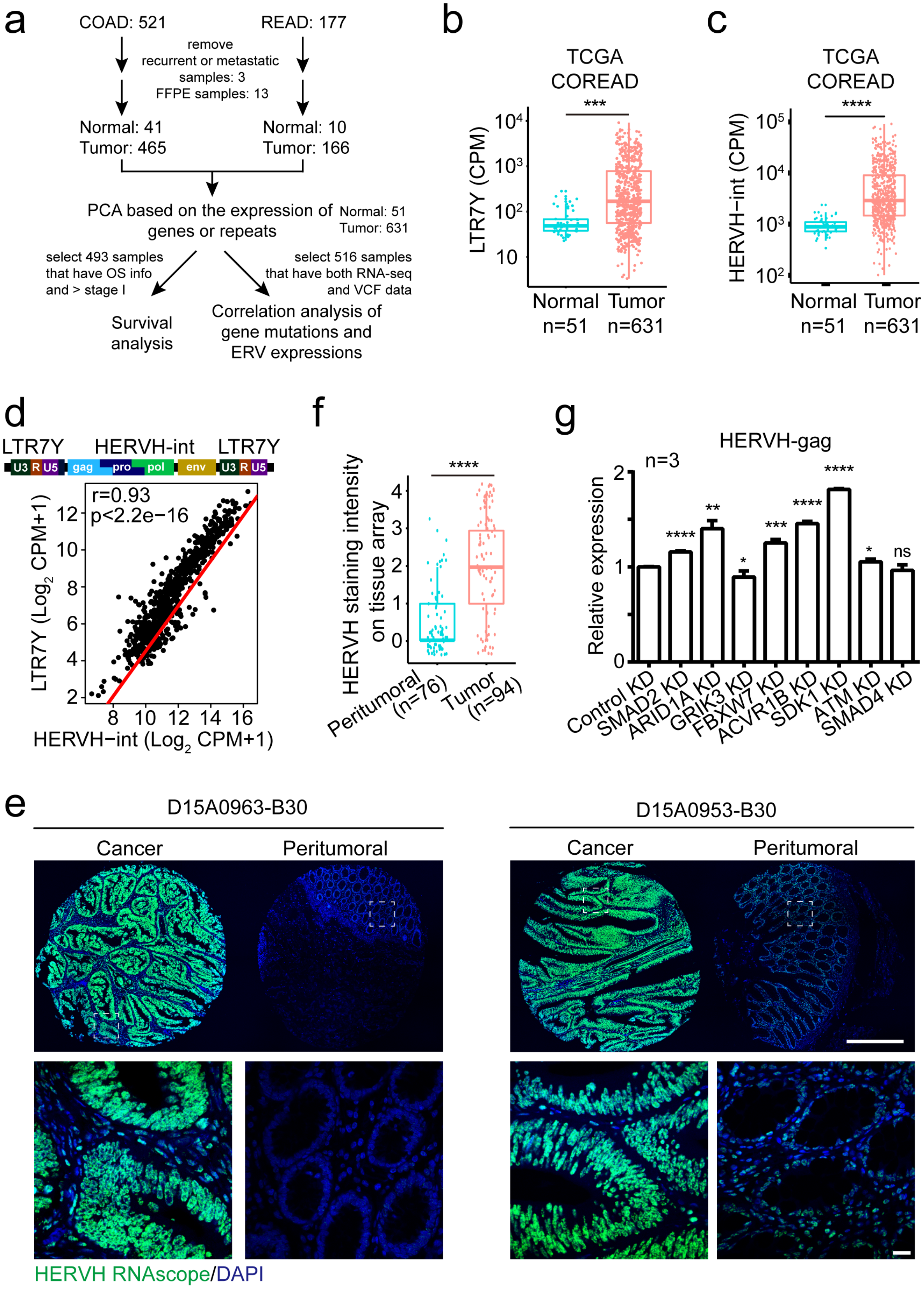
a
COAD: 521
READ: 177
remove
recurrent or metastatic
samples: 3
FFPE samples: 13
Normal: 41
Tumor: 465
Normal: 10
Tumor: 166
PCA based on the expression of
genes or repeats
Normal: 51
Tumor: 631
select 493 samples
that have OS info
and > stage I
select 516 samples
that have both RNA-seq
and VCF data
Survival
analysis
Correlation analysis of
gene mutations and
ERV expressions
b
TCGA
COREAD
LTR7Y (CPM)
***
Normal
n=51
Tumor
n=631
c
TCGA
COREAD
HERVH−int (CPM)
****
Normal
n=51
Tumor
n=631
d
LTR7Y
HERVH-int
LTR7Y
U3 R U5
gag
pro
pol
env
U3 R U5
r=0.93
p<2.2e−16
LTR7Y (Log2 CPM+1)
HERVH−int (Log2 CPM+1)
f
HERVH staining intensity
on tissue array
****
Peritumoral
(n=76)
Tumor
(n=94)
g
HERVH-gag
n=3
Relative expression
Control KD
SMAD2 KD
ARID1A KD
GRIK3 KD
FBXW7 KD
ACVR1B KD
SDK1 KD
ATM KD
SMAD4 KD
ns
e
D15A0963-B30
Cancer
Peritumoral
D15A0953-B30
Cancer
Peritumoral
HERVH RNAscope/DAPI

Supplementary Figure 1. Expression of HERVH in CRC samples. (a) The inclusion and exclusion criteria for the TCGA-COREAD samples used in Figure 1. (b-c) Box plots of the expression of LTR7Y and HERVH-int in the TCGA-COREAD dataset. The box-plot center represents median, the bottom and top lines represent the 25th and 75th percentiles, the whiskers extending to ± 1.5 × interquartile range (IQR). Two-tailed unpaired t test, ***$p < 0.001$, ****$p < 0.0001$. (d) Correlation of the expression of HERVH-int and LTR7Y in the TCGA-COREAD dataset. The Pearson correlation coefficient (r) and the *p*-value are shown (two-tailed test). CPM, counts per million. (e) Representative images of RNAscope staining of HERVH transcripts on CRC tissue array. Bars: 500 μm in the upper panels and 20 μm in lower insets. (f) Quantification of the RNAscope signals from the peritumoral and tumor tissues on the CRC tissue array. The box-plot center represents median, the bottom and top lines represent the 25th and 75th percentiles, the whiskers extending to ± 1.5 × interquartile range (IQR). Two-tailed unpaired t test, ****$p < 0.0001$. (g) qPCR analysis of HERVH expression in cells treated with the indicated siRNAs. KD, knockdown. Data are presented as mean values ± SD from three independent experiments, two-tailed unpaired t test, ns: not significant, *$p < 0.05$, **$p < 0.01$, ***$p < 0.001$, and ****$p < 0.0001$. Source data including exact *p*-values are provided as a Source Data file.

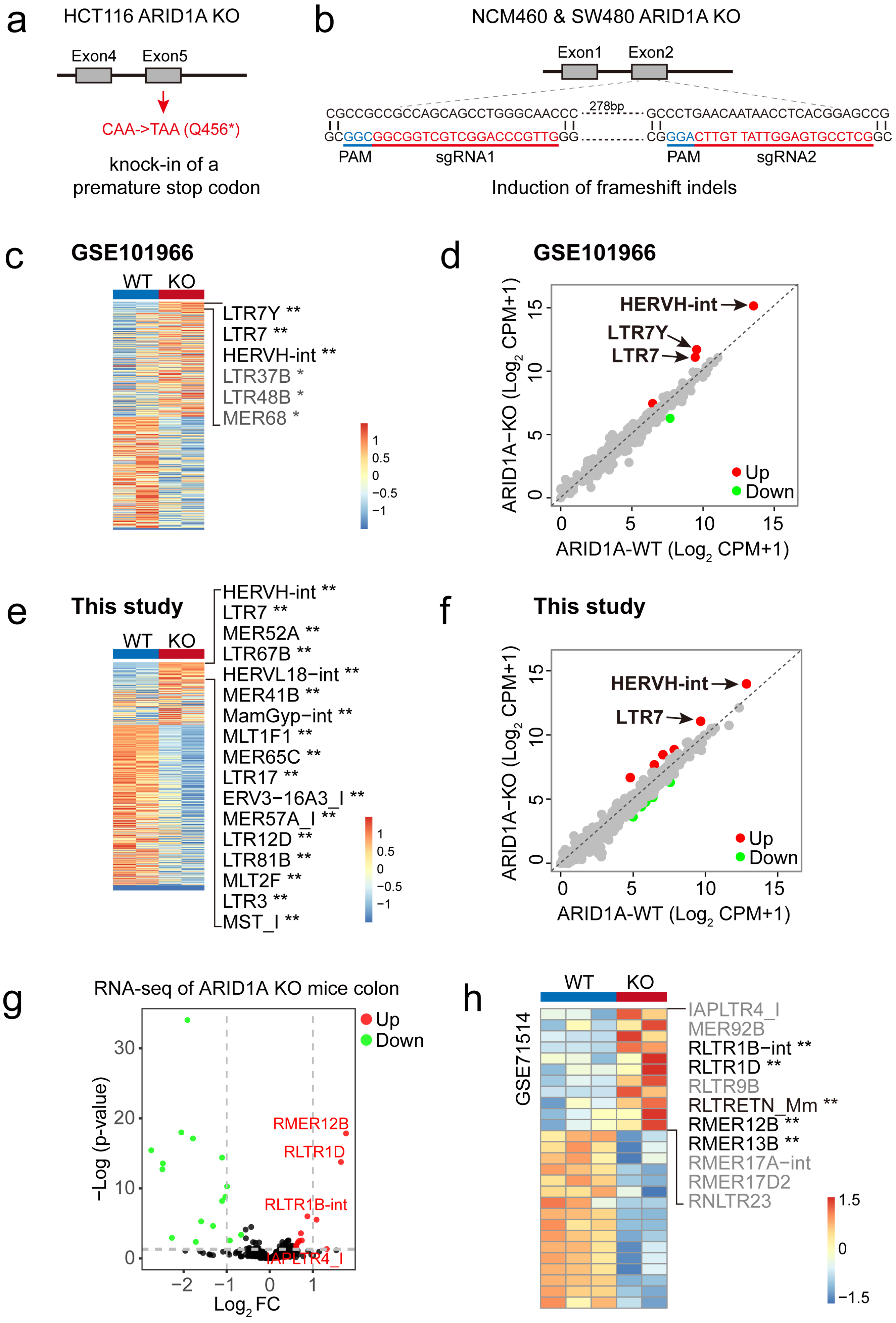
a
HCT116 ARID1A KO
Exon4
Exon5
CAA->TAA (Q456*)
knock-in of a premature stop codon
b
NCM460 & SW480 ARID1A KO
Exon1
Exon2
CGCCGCCGCCAGCAGCCTGGGCAACCC
278bp
GCCCTGAACAATAACCTCACGGAGCCG
GCGGCGGCGGTCGTCGGACCCGTTGGG
CGGGACTTGT TATTGGAGTGCCTCGGC
PAM
sgRNA1
PAM
sgRNA2
Induction of frameshift indels
c
GSE101966
WT
KO
LTR7Y **
LTR7 **
HERVH-int **
LTR37B *
LTR48B *
MER68 *
d
GSE101966
HERVH-int
LTR7Y
LTR7
Up
Down
ARID1A−KO (Log2 CPM+1)
ARID1A-WT (Log2 CPM+1)
e
This study
WT
KO
HERVH-int **
LTR7 **
MER52A **
LTR67B **
HERVL18−int **
MER41B **
MamGyp−int **
MLT1F1 **
MER65C **
LTR17 **
ERV3−16A3_I **
MER57A_I **
LTR12D **
LTR81B **
MLT2F **
LTR3 **
MST_I **
f
This study
HERVH-int
LTR7
Up
Down
ARID1A−KO (Log2 CPM+1)
ARID1A-WT (Log2 CPM+1)
g
RNA-seq of ARID1A KO mice colon
Up
Down
RMER12B
RLTR1D
RLTR1B-int
IAPLTR4_I
−Log (p-value)
Log2 FC
h
GSE71514
WT
KO
IAPLTR4_I
MER92B
RLTR1B−int **
RLTR1D **
RLTR9B
RLTRETN_Mm **
RMER12B **
RMER13B **
RMER17A−int
RMER17D2
RNLTR23

Supplementary Figure 2. ARID1A loss derepresses ERVs. (a-b) Schematics for the strategies of generating ARID1A KO cells. HCT116 ARID1A KO is achieved by knocking-in of a premature stop codon (Q456*), and NCM460 and SW480 ARID1A KO cells are generated by induction of frameshift indels after CRISPR-Cas9 cleavages. (c-f) Heatmaps and scatter plots of the expressions of different ERVs in ARID1A WT and KO HCT116 cells generated with the GSE101966 dataset (c-d) or our own RNA-seq results (e-f). The differential expression is tested based on a model using the negative binomial distribution by DESeq2 (version v1.22.2, with two-tailed likelihood ratio test). The adjusted *p*-values are labeled as $*p < 0.05$, $**p < 0.01$. The up- or downregulated ERVs in ARID1A KO cells are determined using cut-off values of $|\text{Log}_2\ \text{FC}| > 1$ and adjusted *p*-value < 0.05. (g) Volcano plot of differentially expressed ERVs in WT and ARID1A KO mice colon analyzed using DESeq2 (version v1.22.2, with two-tailed likelihood ratio test). Up (red) and down (green) regulated ERVs are determined with the cut-off values of adjusted *p*-value < 0.05 and $|\text{Log}_2\ \text{FC}| > 0.585$. (h) Heatmap generated with the GSE71514 dataset showing differentially expressed mouse ERVs in ARID1A KO colons analyzed using DESeq2 (version v1.22.2, with two-tailed likelihood ratio test). The adjusted *p*-values are labeled as $**p < 0.01$. The source data including exact *p*-values are provided as a Source Data file.

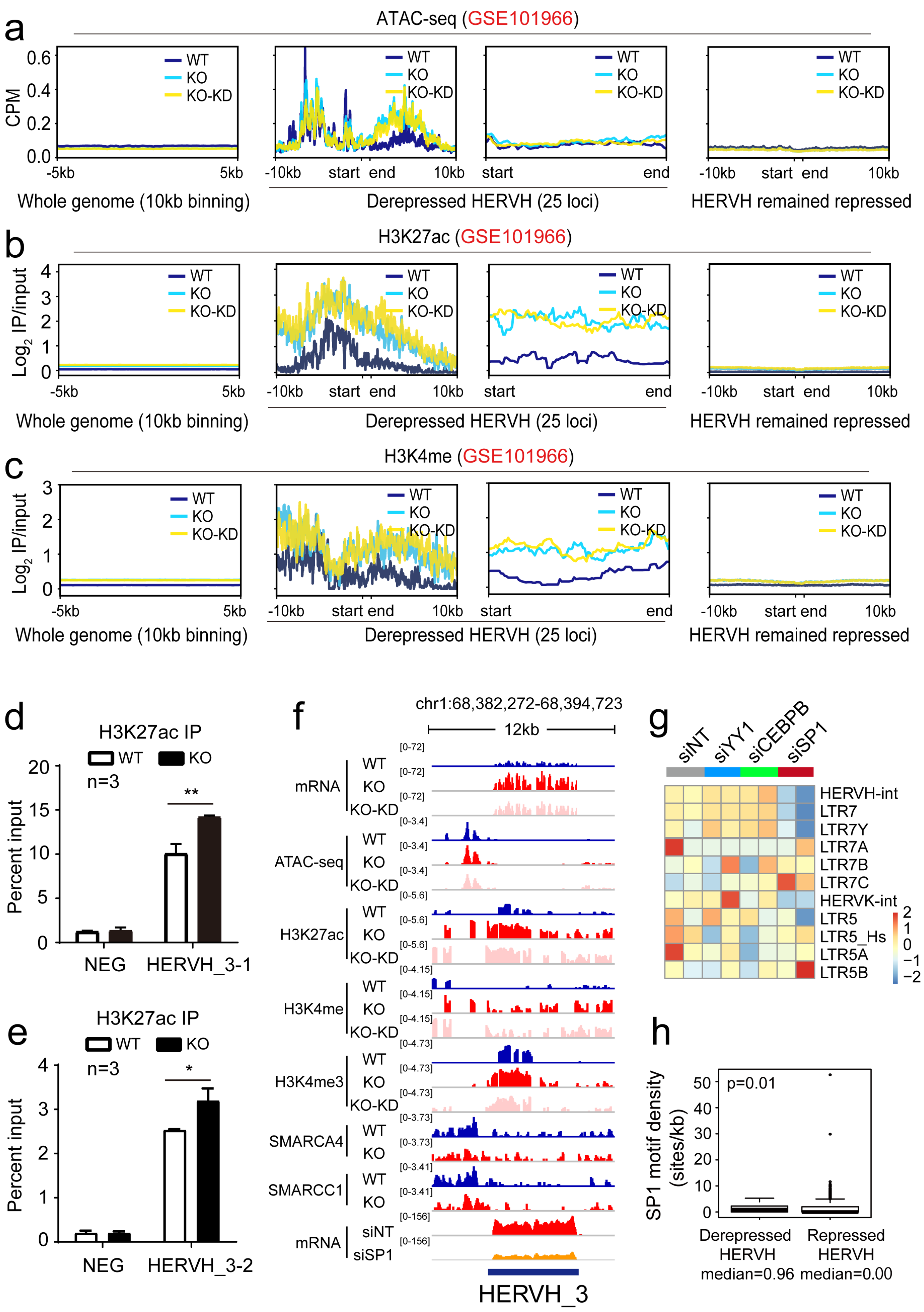

a
ATAC-seq (GSE101966)
CPM
WT
KO
KO-KD
-5kb
5kb
Whole genome (10kb binning)
-10kb start end 10kb
start end
Derepressed HERVH (25 loci)
HERVH remained repressed
b
H3K27ac (GSE101966)
Log2 IP/input
c
H3K4me (GSE101966)
d
H3K27ac IP
n=3
Percent input
NEG
HERVH_3-1
e
HERVH_3-2
f
chr1:68,382,272-68,394,723
12kb
mRNA
ATAC-seq
H3K27ac
H3K4me
H3K4me3
SMARCA4
SMARCC1
siNT
siSP1
HERVH_3
g
siNT
siYY1
siCEBPB
siSP1
HERVH-int
LTR7
LTR7Y
LTR7A
LTR7B
LTR7C
HERVK-int
LTR5
LTR5_Hs
LTR5A
LTR5B
h
p=0.01
SP1 motif density (sites/kb)
Derepressed HERVH
median=0.96
Repressed HERVH
median=0.00

Supplementary Figure 3. The histone modifications and transcription factor involved in HERVH derepression. (a-c) Averaged signals of ATAC-seq and ChIP-seq with H3K27ac or H3K4me antibodies around or at the derepressed *HERVH* loci. Signals from the whole genome (10 kb bins) and the rest *HERVH* loci that remain repressed are shown for comparisons. WT: wild type HCT116 cells, KO: ARID1A KO HCT116 cells, KO-KD: ARID1A KO cells with ARID1B shRNA knockdown. (d-e) ChIP-qPCR with two different primer sets confirming the increased H3K27ac at the *HERVH_3* locus upon ARID1A loss. Data are presented as mean values ± SD from three independent experiments, two-tailed unpaired t test, $*p < 0.05$, $**p < 0.01$. (f) Genomic snapshot of the signals from RNA-seq, ATAC-seq, and ChIP-seq with the indicated antibodies at a representative *HERVH* locus. (g) Heatmap showing the transcripts abundance of HERVH (HERVH-int and LTR7s) and HERVK (HERVK-int and LTR5s) upon knockdown of the indicated transcription factors by siRNAs in ARID1A KO HCT116 cells. (h) The derepressed *HERVH* loci are predicted to harbor more SP1 binding motifs than the rest *HERVH* loci that remain repressed. Data are presented as mean values ± SD, Wilcoxon rank sum test (one-tailed), $**p = 0.01$. Source data including exact *p*-values are provided as a Source Data file.

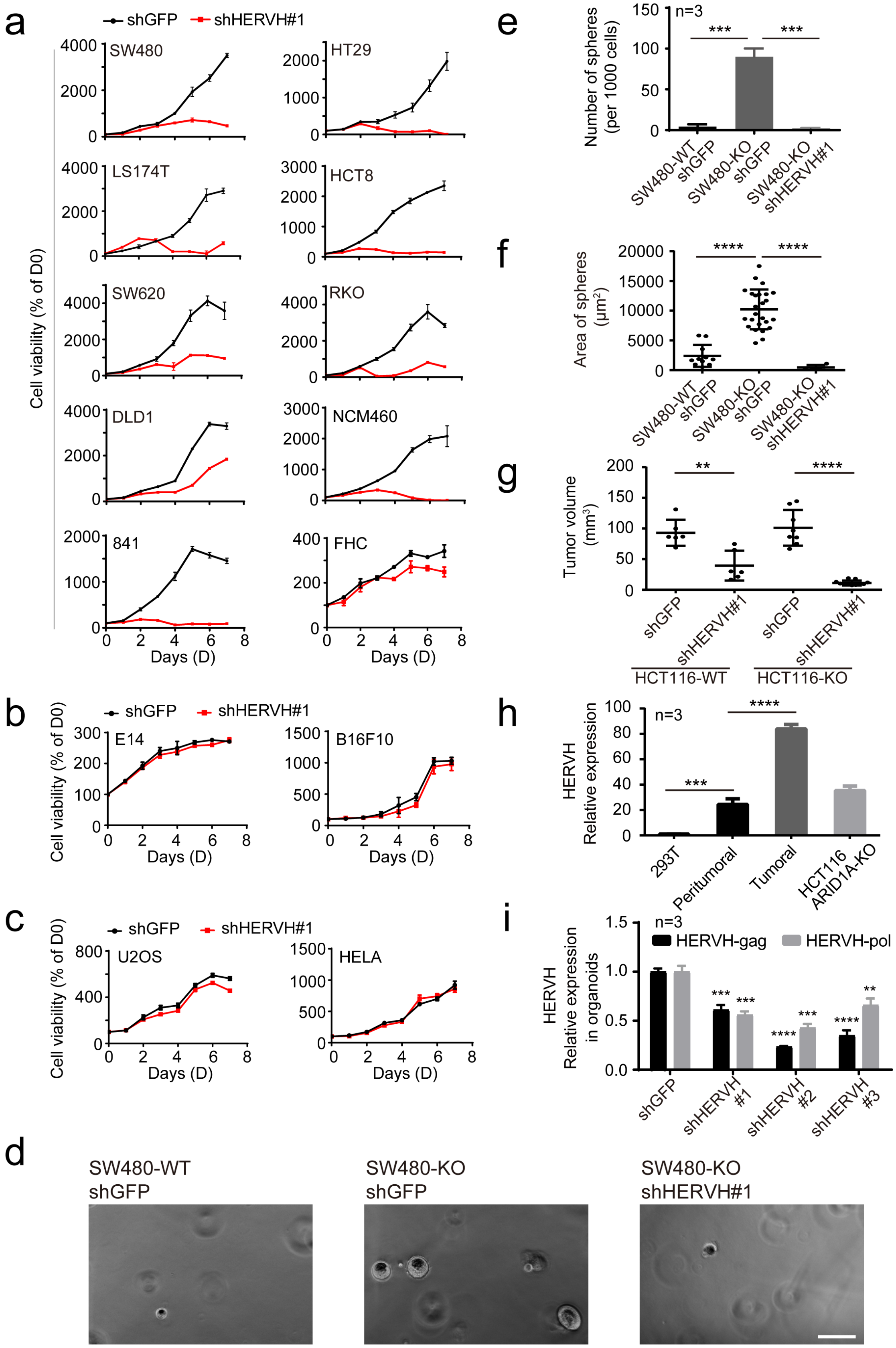

a
shGFP
shHERVH#1
Cell viability (% of D0)
SW480
HT29
LS174T
HCT8
SW620
RKO
DLD1
NCM460
841
FHC
Days (D)
b
E14
B16F10
c
U2OS
HELA
d
SW480-WT shGFP
SW480-KO shGFP
SW480-KO shHERVH#1
e
n=3
Number of spheres (per 1000 cells)
f
Area of spheres (μm²)
g
Tumor volume (mm³)
HCT116-WT
HCT116-KO
h
HERVH Relative expression
293T
Peritumoral
Tumoral
HCT116 ARID1A-KO
i
HERVH-gag
HERVH-pol
HERVH Relative expression in organoids
shGFP
shHERVH #1
shHERVH #2
shHERVH #3

Supplementary Figure 4. HERVH is vital for the growth of CRC cells. (a) The viability of the indicated colorectal cell lines treated with shRNA targeting control GFP or HERVH. Data presented are from at least three independent experiments. (b) Mice embryonic stem cells (E14) and mice melanoma cells (B16F10) that do not have HERVH elements are insensitive to HERVH knockdown. Data presented are from at least three independent experiments. (c) The viability of osteosarcoma cells (U2OS) and cervical cancer cells (HELA) after treatment with shRNA targeting control GFP or HERVH. Data presented are from at least three independent experiments. (d) Representative brightfield images showing that knockdown of HERVH inhibits sphere formation of SW480 ARID1A KO cells. Results are representative of three independent experiments. Bar: 130 μm. (e-f) Quantifications of sphere number and sphere size of the indicated experimental groups. Data are presented as mean values ± SD from three independent experiments, two-tailed unpaired t test, $***p < 0.001$, $****p < 0.0001$. (g) HERVH knockdown suppresses tumor growth of WT and ARID1A KO HCT116 cells in mouse subcutaneous xenograft tumor models. 6 mice each were used for control and HERVH KD WT HCT116 cells. 8 and 11 mice were used for control and HERVH KD ARID1A KO cells respectively. Data are presented as mean values ± SD, two-tailed unpaired t test, $**p < 0.01$, $****p < 0.0001$. (h) qPCR analysis of HERVH expression in patient samples used to establish the CRC organoids. Data are presented as mean values ± SD from three independent experiments, two-tailed unpaired t test, $***p < 0.001$, $****p < 0.0001$. (i) The knockdown efficiency of different HERVH shRNAs in organoids examined by qPCR. Data are presented as mean values ± SD from three independent experiments, two-tailed unpaired t test, $**p < 0.01$, $***p < 0.001$, and $****p < 0.0001$. Source data including exact *p*-values are provided as a Source Data file.

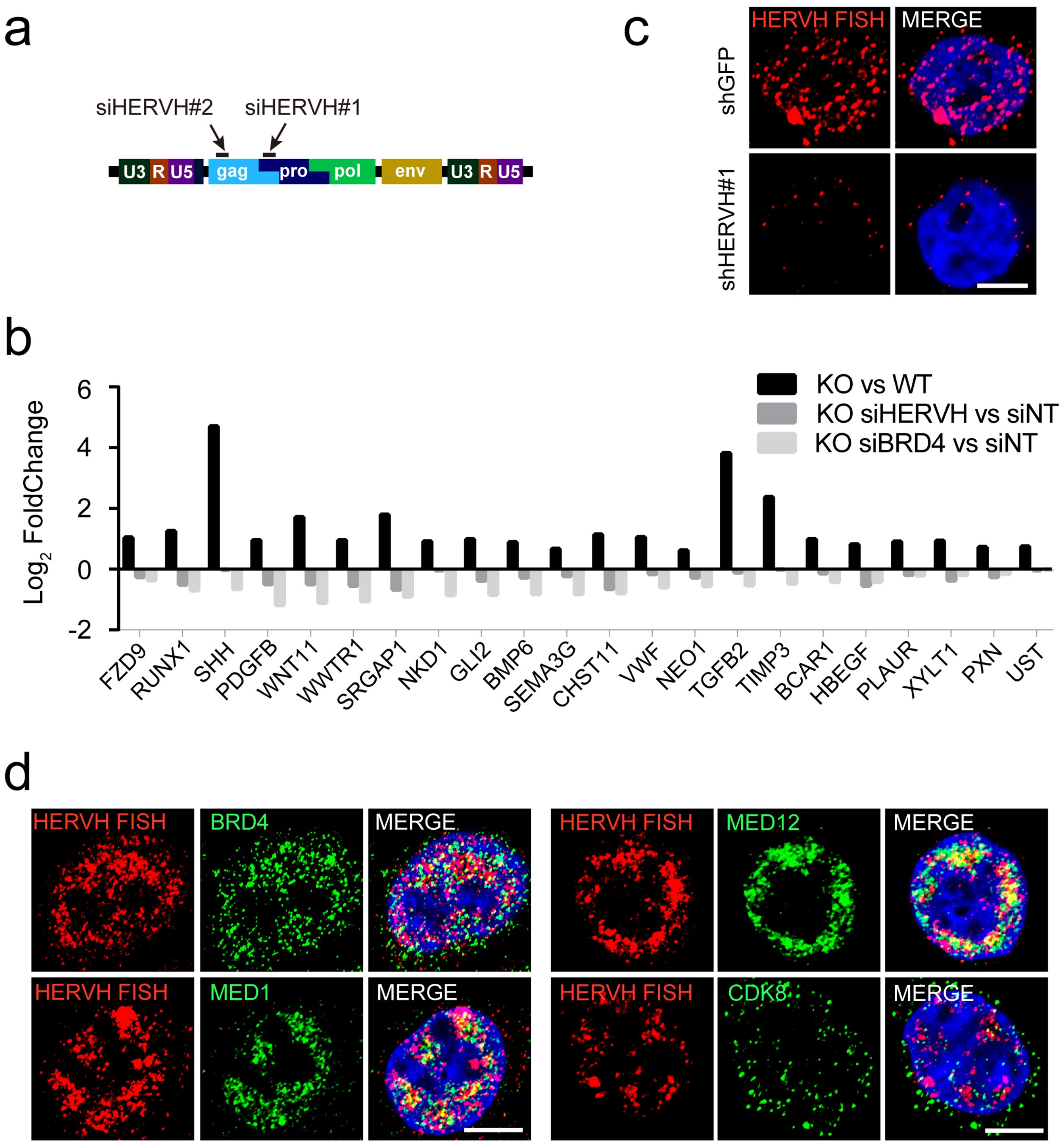

Supplementary Figure 5. Partial colocalization of HERVH RNA with components of the mediator complex. (a) Schematic showing the siRNA targeted regions on HERVH. (b) The FC of a group of representative genes whose expression increases in ARID1A KO cells but decreases upon knockdown of HERVH or BRD4. (c) Validation of the specificity of HERVH FISH signals. Results are representative of three independent experiments. Bar: 5 μm. (d) Representative immunofluorescence images showing different degrees of colocalization between HERVH transcripts (red) and endogenous BRD4 or other components in the mediator complex (green). Results are representative of three independent experiments. Bars: 5 μm. Source data are provided as a Source Data file.

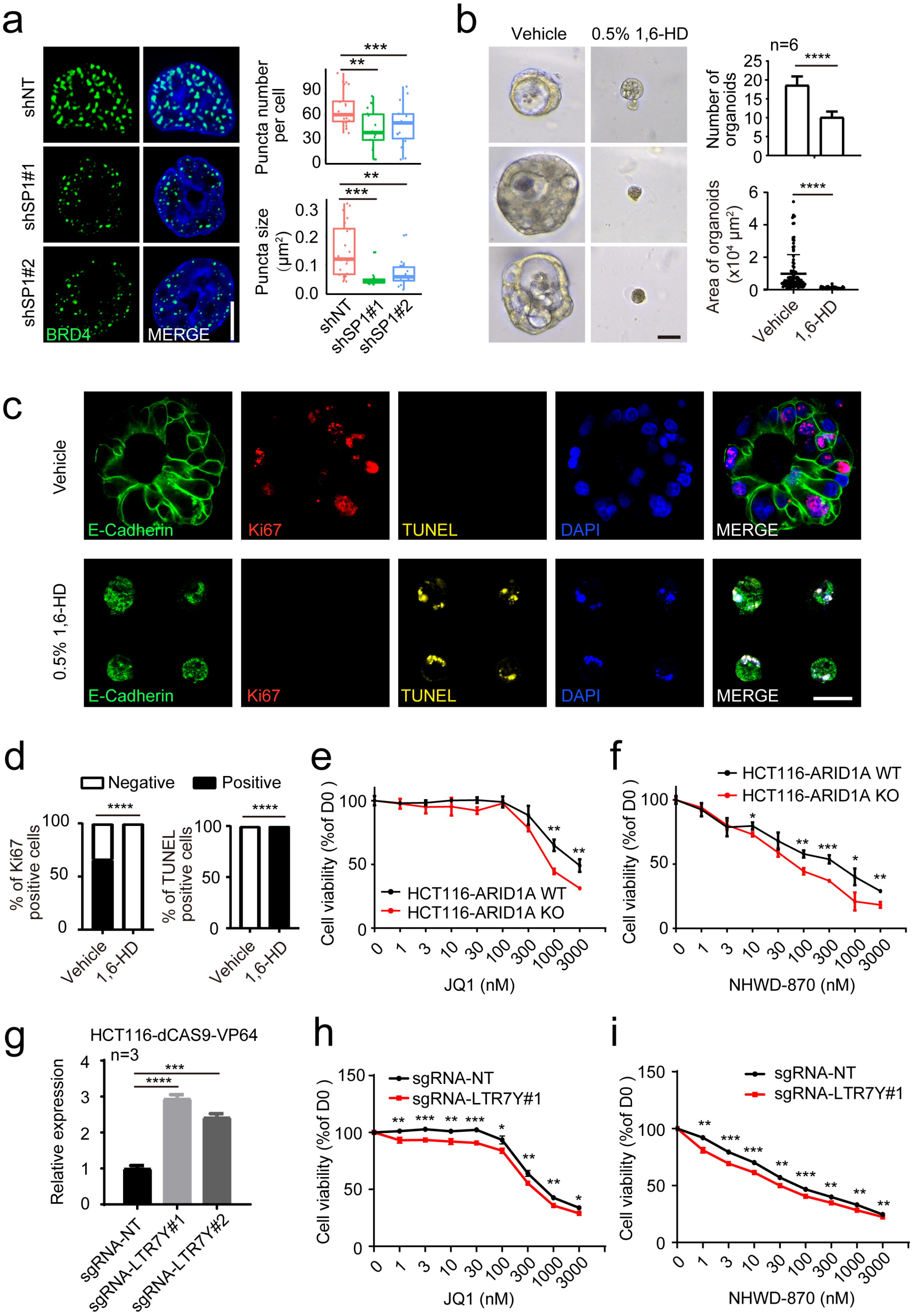
a
shNT
shSP1#1
shSP1#2
BRD4
MERGE
Puncta number per cell
Puncta size (μm²)
shNT
shSP1#1
shSP1#2
b
Vehicle
0.5% 1,6-HD
n=6
Number of organoids
Area of organoids (x10⁴ μm²)
Vehicle
1,6-HD
c
Vehicle
0.5% 1,6-HD
E-Cadherin
Ki67
TUNEL
DAPI
MERGE
d
Negative
Positive
% of Ki67 positive cells
% of TUNEL positive cells
Vehicle
1,6-HD
e
Cell viability (%of D0)
HCT116-ARID1A WT
HCT116-ARID1A KO
JQ1 (nM)
f
HCT116-ARID1A WT
HCT116-ARID1A KO
NHWD-870 (nM)
g
HCT116-dCAS9-VP64
n=3
Relative expression
sgRNA-NT
sgRNA-LTR7Y#1
sgRNA-LTR7Y#2
h
sgRNA-NT
sgRNA-LTR7Y#1
JQ1 (nM)
i
sgRNA-NT
sgRNA-LTR7Y#1
NHWD-870 (nM)

Supplementary Figure 6. Disrupting the BRD4 puncta by 1,6-HD inhibits the proliferation of CRC organoids. (a) Representative images and quantifications of GFP-BRD4 nuclear foci in control (shNT, n=21), shSP1#1 (n=15), or shSP1#2 (n=14) treated cells. The box-plot center represents median, the bottom and top lines represent the 25th and 75th percentiles, the whiskers extending to $\pm$ 1.5 $\times$ interquartile range (IQR). Data presented are from three independent experiments, two-tailed unpaired t test, $^{**}p < 0.01$, $^{***}p < 0.001$. Bar: 5 μm. (b) Representative brightfield images and quantifications of the number and size of CRC organoids in control vehicle or 0.5% 1,6-HD treated groups. Bar: 50 μm. Data are presented as mean values $\pm$ SD from three independent experiments, two-tailed unpaired t test, $^{****}p < 0.0001$. (c) Representative immunofluorescent images of vehicle or 0.5% 1,6-HD treated CRC organoids stained with E-Cadherin (green), Ki67 (red), TUNEL (yellow), and DAPI (blue). Results are representative of three independent experiments. Bar: 50 μm. (d) Percentages of Ki67 or TUNEL positive cells in control vehicle or 0.5% 1,6-HD treated CRC organoids. Data presented are from three independent experiments, $^{****}p < 0.0001$ by chi-squared test. (e-f) Viability of the ARID1A WT or KO HCT116 cells treated with two different BET inhibitors. Data are presented as mean values $\pm$ SD from three independent experiments, two-tailed unpaired t test, $^{*}p < 0.05$, $^{**}p < 0.01$, and $^{***}p < 0.001$. (g) qPCR analysis of HERVH expression in WT HCT116 cells after endogenous HERVH activation by the CRISPRa SAM system using two different sgRNAs targeting the LTR7Y. Data are presented as mean values $\pm$ SD from three independent experiments, two-tailed unpaired t test, $^{***}p < 0.001$, $^{****}p < 0.0001$. (h-i) Viability of the HERVH-activated cells treated with the two BET inhibitors. Data are presented as mean values $\pm$ SD from three independent experiments, two-tailed unpaired t test, $^{*}p < 0.05$, $^{**}p < 0.01$, and $^{***}p < 0.001$. Source data including exact *p*-values are provided as a Source Data file.

List of Supplementary Data
Supplementary Data 1. Derepression of TEs in CRC.
Supplementary Data 2. ARID1A loss derepresses ERVs.
Supplementary Data 3. ARID1B activates HERVH in the absence of ARID1A.
Supplementary Data 4. The histone modifications and TF involved in HERVH derepression.
Supplementary Data 5. HERVH and BRD4 co-regulated genes.
Supplementary Data 6. HERVH Stellaris FISH Probes.
Supplementary Data 7. Oligonucleotide sequences.
Supplementary Data 8. Knockdown efficiencies.
Supplementary Data 9. Antibodies.
Supplementary Data 10. HERVH consensus sequence for RNAscope probe design.
