# Supplementary material for: ARID1A loss derepresses a group of human endogenous retrovirus-H loci to modulate BRD4-dependent transcription
Source: Nat Commun. 2022 Jun 17;13:3501. doi: 10.1038/s41467-022-31197-4 (PMC9205910; doi:10.1038/s41467-022-31197-4)
Supplement: Supplementary file 3 — Description of additional Supplementary File [file 41467_2022_31197_MOESM3_ESM.pdf]

<u>Descriptions of additional Supplementary Files</u>

Supplementary Data 1 : Derepression of TEs in CRC.

Supplementary Data 2: ARID1A loss derepresses ERVs.

Supplementary Data 3: ARID1B activates HERVH in the absence of ARID1A.

Supplementary Data 4: The histone modifications and TF involved in HERVH derepression.

Supplementary Data 5: HERVH and BRD4 co-regulated genes.

Supplementary Data 6: HERVH Stellaris FISH Probes.

Supplementary Data 7: Oligonucleotide sequences.

Supplementary Data 8: Knockdown efficiencies.

Supplementary Data 9: Antibodies.

Supplementary Data 10: HERVH consensus sequence for RNAscope probe design.
