# Supplementary material for: ARID1A loss derepresses a group of human endogenous retrovirus-H loci to modulate BRD4-dependent transcription
Source: Nat Commun. 2022 Jun 17;13:3501. doi: 10.1038/s41467-022-31197-4 (PMC9205910; doi:10.1038/s41467-022-31197-4)
Supplement: Supplementary file 14 — Reporting Summary [file 41467_2022_31197_MOESM14_ESM.pdf]

nature portfolio

Corresponding author(s): Kai Yuan

Last updated by author(s): 2022-4-27

Reporting Summary

Nature Portfolio wishes to improve the reproducibility of the work that we publish. This form provides structure for consistency and transparency in reporting. For further information on Nature Portfolio policies, see our Editorial Policies and the Editorial Policy Checklist.

Statistics

For all statistical analyses, confirm that the following items are present in the figure legend, table legend, main text, or Methods section.

n/a	Confirmed	
☐	☒	The exact sample size (*n*) for each experimental group/condition, given as a discrete number and unit of measurement
☐	☒	A statement on whether measurements were taken from distinct samples or whether the same sample was measured repeatedly
☐	☒	The statistical test(s) used AND whether they are one- or two-sided<br>*Only common tests should be described solely by name; describe more complex techniques in the Methods section.*
☒	☐	A description of all covariates tested
☐	☒	A description of any assumptions or corrections, such as tests of normality and adjustment for multiple comparisons
☐	☒	A full description of the statistical parameters including central tendency (e.g. means) or other basic estimates (e.g. regression coefficient) AND variation (e.g. standard deviation) or associated estimates of uncertainty (e.g. confidence intervals)
☐	☒	For null hypothesis testing, the test statistic (e.g. *F*, *t*, *r*) with confidence intervals, effect sizes, degrees of freedom and *P* value noted<br>*Give P values as exact values whenever suitable.*
☒	☐	For Bayesian analysis, information on the choice of priors and Markov chain Monte Carlo settings
☒	☐	For hierarchical and complex designs, identification of the appropriate level for tests and full reporting of outcomes
☐	☒	Estimates of effect sizes (e.g. Cohen's *d*, Pearson's *r*), indicating how they were calculated

Our web collection on statistics for biologists contains articles on many of the points above.

Software and code

Policy information about availability of computer code

Data collection	qPCR was performed in a Quantstudio 3 Real-Time PCR Instrument.<br>Immunofluorescence images were collected by LSM880 confocal microscope (Zeiss).<br>Western Blot images were collected by ChemiDoc XRS+ system (Bio-Rad).
Data analysis	1) RNA-seq analysis: Raw reads were first cleaned using trim_galore v0.6.0 (http://www.bioinformatics.babraham.ac.uk/projects/trim_galore/) with default parameters. The reads from each RNA-seq sample were then mapped to hg38 or mm9 genome assembly downloaded from UCSC, using STAR v2.5.3a. Genes expression was quantified using featureCounts v1.6.5 of subread-1.6.5 package based on hg38 RefSeq genes annotation file. Principal component analysis was conducted with the functions “vst” and “plotPCA” from R package DESeq2 v1.22.2. The heatmap of differentially expressed genes or repeats was created using R package pheatmap v1.0.12. The KEGG enrichment analysis was performed using the function “enrichKEGG” from the R package clusterProfiler v3.10.1. Venn diagrams were prepared with the R package Vennerable 3.1.0.9000 and venn 1.10.<br>2) The survival curves of the two groups were compared using log-rank test from the function “survdiff” in R package survival v2.44-1.1.<br>3) ATAC-seq and ChIP-seq analyses: The reads were aligned to the hg38 genome assembly using STAR v2.5.3a. Duplicate reads were then removed using MarkDuplicates from gatk package v.4.1.4.1. Replicate samples were merged using the samtools v1.10. Bigwig tracks were generated using bamCoverage from python package deeptools 3.3.1. ATAC-seq and ChIP-seq profiles were created by computeMatrix and plotProfile in deeptools 3.3.1. IGV v.2.4.13 was used to visualize the bigwig tracks.<br>The images were analyzed and measured with ZEN 2.3 blue edition (Zeiss) and Image J (v1.48).<br>All statistical and correlation analyses were performed using GraphPad Prism(v8.0).

For manuscripts utilizing custom algorithms or software that are central to the research but not yet described in published literature, software must be made available to editors and reviewers. We strongly encourage code deposition in a community repository (e.g. GitHub). See the Nature Portfolio guidelines for submitting code & software for further information.

Data

Policy information about availability of data

All manuscripts must include a data availability statement. This statement should provide the following information, where applicable:

- Accession codes, unique identifiers, or web links for publicly available datasets
- A description of any restrictions on data availability
- For clinical datasets or third party data, please ensure that the statement adheres to our policy

1.publicly available datasets
(1)The TCGA datasets used in this study, including the RNA-seq BAM files, the gene raw count data (htseq-count files), and the annotated somatic simple nucleotide variation files (MuTect2 VCF) of patients with colon adenocarcinoma (COAD) and rectum adenocarcinoma (READ), were accessed through dbGaP accession number phs000178.v11.p8.
(2)GSE50760, GSE71514, GSE101966, and the colorectal cancer cell lines in CCLE (PRJNA523380)

2.RNA-seq generated in this study
GSE180475

Field-specific reporting

Please select the one below that is the best fit for your research. If you are not sure, read the appropriate sections before making your selection.

[x] Life sciences [ ] Behavioural & social sciences [ ] Ecological, evolutionary & environmental sciences

For a reference copy of the document with all sections, see nature.com/documents/nr-reporting-summary-flat.pdf

Life sciences study design

All studies must disclose on these points even when the disclosure is negative.

Sample size	No statistical methods were used to predetermine the sample sized. Three or more sample sizes were chosen to provide enough replicates for statistical analysis, and the exact sample size of each experiment was determined by availability of biological samples. Sample size and statistical analysis are provided in figure legend and Source Data file.
Data exclusions	For TCGA-COREAD patients tissues sample, paraffin-embedded (FFPE) tissue samples were removed, as RNA obtained from FFPE tissue samples is usually degraded, fragmented and chemically modified, resulting in unsatisfactory sequencing data.
Replication	All experiments were conducted at least three times independently, and all attempts at replication were successful.
Randomization	The mice were randomly distributed into groups, and then inoculated with different tumor cells. All the cells or organoids were cultured in the same environment with the same density, and were allocated into experimental groups randomly.
Blinding	The investigators were blinded during tumor measurements, tumor size analyses, and quantification of RNAscope staining of HERVH transcripts on CRC tissue array. For all the cell biological experiments, investigators were not blinded to group allocation for data collection and analysis since the same investigator designed and performed the experiments.

Reporting for specific materials, systems and methods

We require information from authors about some types of materials, experimental systems and methods used in many studies. Here, indicate whether each material, system or method listed is relevant to your study. If you are not sure if a list item applies to your research, read the appropriate section before selecting a response.

Materials & experimental systems

n/a	Involved in the study	
[ ]	[x]	Antibodies
[ ]	[x]	Eukaryotic cell lines
[x]	[ ]	Palaeontology and archaeology
[ ]	[x]	Animals and other organisms
[ ]	[x]	Human research participants
[x]	[ ]	Clinical data
[x]	[ ]	Dual use research of concern

Methods

n/a	Involved in the study	
[x]	[ ]	ChIP-seq
[x]	[ ]	Flow cytometry
[x]	[ ]	MRI-based neuroimaging

Antibodies

Antibodies used	Antibody used for Immunofluorescence: rabbit anti-E-Cadherin (1:400, CST, 3195S), mouse anti-Ki67 (1:400, CST, 9449S), rabbit anti-BRD4 (1:500, active motif, 39909), rabbit anti-MED1 (1:500, Abcam, ab64965), anti-MED12 (1:500, Bethyl, A300-774A), anti-CDK8 (1:500, Active Motif, 61481),Goat anti-Rabbit IgG (H+L) Cross-Adsorbed Secondary Antibody, Alexa FluorTM488 (1:500, Thermo Fisher Scientific, A11008),Goat anti-Rabbit IgG (H+L) Cross-Adsorbed Secondary Antibody, Alexa FluorTM647 (1:400, Thermo Fisher Scientific, A21244), Goat anti-Mouse IgG (H+L) Cross-Adsorbed Secondary Antibody, Alexa FluorTM568 (1:400, Thermo Fisher Scientific, A11004).<br>Antibody used for ChIP: anti-ARID1A (12354S, CST), anti-ARID1B (sc-32762 X, Santa Cruz), anti-SMARCA4 (ab110641, Abcam), anti-H3K27ac (ab4729, Abcam).<br>Antibody used for western blot: anti-ARID1A (1:1000, ab182560, Abcam), anti-BRD4 (1:1000, 39909, active motif), anti-Tubulin (1:3000, 3873s, CST), Goat anti Rabbit IgG (H+L) Secondary Antibody (1:5000, Thermo Fisher Scientific, 31460), Goat anti Mouse IgG (H+L) Secondary Antibody (1:5000, Thermo Fisher Scientific, 31430).
Validation	The antibodies were purchased from commercial sources, and validated by the manufacturers.<br>anti-E-Cadherin (3195S,CST), https://www.cellsignal.com/products/primary-antibodies/e-cadherin-24e10-rabbit-mab/3195,<br>anti-Ki67 (9449S, CST), https://www.cellsignal.com/products/primary-antibodies/ki-67-8d5-mouse-mab/9449,<br>anti-BRD4 (39909,active motif), https://www.activemotif.com/catalog/details/39909/brd4-antibody-pab,<br>anti-MED1 (ab64965,Abcam), https://www.abcam.com/trap220med1-antibody-ab64965.html,<br>anti-MED12 (A300-774A,Bethyl), https://www.thermofisher.cn/cn/zh/antibody/product/MED12-Antibody-Polyclonal/A300-774A,<br>anti-CDK8 (61481,active motif), https://www.activemotif.com/catalog/details/61481/cdk8-antibody-pab,<br>anti-ARID1A (12354S, CST), https://www.cellsignal.com/products/primary-antibodies/arid1a-baf250a-d2a8u-rabbit-mab/12354,<br>anti-ARID1B (sc-32762 X,Santa Cruz), https://www.scbt.com/p/arid1b-antibody-kmn1,https://doi.org/10.1371/journal.pgen.1005748,<br>anti-SMARCA4 (ab110641,Abcam), https://www.abcam.com/BRG1-antibody-EPNCIR111A-ab110641.html, doi: 10.1038/ng.3744.<br>H3K27ac (ab4729,Abcam), https://www.abcam.com/histone-h3-acetyl-k27-antibody-chip-grade-ab4729.html,<br>anti-ARID1A (ab182560, Abcam), https://www.abcam.cn/ARID1A-antibody-EPR13501-ab182560.html,<br>anti-Tubulin (3873s,CST), https://www.cellsignal.com/products/primary-antibodies/a-tubulin-dm1a-mouse-mab/3873.

Eukaryotic cell lines

Policy information about cell lines

Cell line source(s)	SW480, LS174T, SW620, HT29, HCT8 and RKO cells were kindly provided by Fu kai (IMPM, Central South University) and Joong Sup Shim (University of Macau). B16F10 was kindly provided by Xiang Chen (Central South University). The original commercial source of the above cells was ATCC. HCT116, DLD1, CRL1790/841, E14, HELA, U2OS, NCM460 and 293T cells were purchased from ATCC. HCT116 ARID1A KO cell line was purchased from Horizon Discovery.
Authentication	All the cell lines were routinely authenticated by morphology check using microscope.
Mycoplasma contamination	All cell lines were tested to be mycoplasma negative.
Commonly misidentified lines<br>(See ICLAC register)	No commonly misidentified cell lines were used in this study.

Animals and other organisms

Policy information about studies involving animals; ARRIVE guidelines recommended for reporting animal research

Laboratory animals	The 4-5 weeks old female BALB/c nude mice were purchased from Hunan SJA Laboratory Animal Co., Ltd. (Changsha, China). All the mice were housed under the SPF environment with a 12h light-dark cycle, and had a temperature of 22-24℃ with 50–60% humidity.
Wild animals	No wild animals were used in the study.
Field-collected samples	No field-collected samples were used in the study.
Ethics oversight	All the animal experiments were approved by the Medical Ethics Committee of Central South University, and conducted according to the Guidelines of Animal Handling and Care in Medical Research in Hunan Province, China.

Note that full information on the approval of the study protocol must also be provided in the manuscript.

Human research participants

Policy information about studies involving human research participants

Population characteristics	A 63-year-old man with rectal adenocarcinoma (AJCC stage IIA) (MSS: MSH2+, MSH6+, MLH1+, PMS2+)
Recruitment	Colorectal cancer samples were collected by physicians after signing an informed consent at the third Xiangya Hospital, only previously untreated colorectal cancer samples were included. There was no potential self-selection bias.
Ethics oversight	All the human tissue related experiments were approved by the Medical Ethics Committee of Central South University, and

the informed consent was obtained from the patients.

Note that full information on the approval of the study protocol must also be provided in the manuscript.
